# Investigation of the Effect of ECAP Parameters on Hardness, Tensile Properties, Impact Toughness, and Electrical Conductivity of Pure Cu through Machine Learning Predictive Models

**DOI:** 10.3390/ma15249032

**Published:** 2022-12-17

**Authors:** Mahmoud Shaban, Mohammed F. Alsharekh, Fahad Nasser Alsunaydih, Abdulrahman I. Alateyah, Majed O. Alawad, Amal BaQais, Mokhtar Kamel, Ahmed Nassef, Medhat A. El-Hadek, Waleed H. El-Garaihy

**Affiliations:** 1 Department of Electrical Engineering, College of Engineering, Qassim University, Unaizah 56452, Saudi Arabia; 2 Department of Electrical Engineering, Faculty of Engineering, Aswan University, Aswan 81542, Egypt; 3 Department of Mechanical Engineering, College of Engineering, Qassim University, Unaizah 56452, Saudi Arabia; 4 Materials Science Research Institute, King Abdulaziz City for Science and Technology (KACST), Riyadh 12354, Saudi Arabia; 5 Department of Chemistry, College of Science, Princess Nourah bint Abdulrahman University, Riyadh 11671, Saudi Arabia; 6 Mechanical Engineering Department, Faculty of Engineering, Suez Canal University, Ismailia 41522, Egypt; 7 Production Engineering and Mechanical Engineering Department, Faculty of Engineering, Port Said University, Port Fouad 42534, Egypt; 8 President of East Port Said University of Technology, North Sinai 45632, Egypt

**Keywords:** ECAP parameters, Cu and related alloys, mechanical properties, electrical conductivity, machine learning, ANN

## Abstract

Copper and its related alloys are frequently adopted in contemporary industry due to their outstanding properties, which include mechanical, electrical, and electronic applications. Equal channel angular pressing (ECAP) is a novel method for producing ultrafine-grained or nanomaterials. Modeling material design processes provides exceptionally efficient techniques for minimizing the efforts and time spent on experimental work to manufacture Cu or its associated alloys through the ECAP process. Although there have been various physical-based models, they are frequently coupled with several restrictions and still require significant time and effort to calibrate and enhance their accuracies. Machine learning (ML) techniques that rely primarily on data-driven models are a viable alternative modeling approach that has recently achieved breakthrough achievements. Several ML algorithms were used in the modeling training and testing phases of this work to imitate the influence of ECAP processing parameters on the mechanical and electrical characteristics of pure Cu, including the number of passes (N), ECAP die angle (φ), processing temperature, and route type. Several experiments were conducted on pure commercial Cu while altering the ECAP processing parameters settings. Linear regression, regression trees, ensembles of regression trees, the Gaussian process, support vector regression, and artificial neural networks are the ML algorithms used in this study. Model predictive performance was assessed using metrics such as root-mean-squared errors and R^2^ scores. The methodologies presented here demonstrated that they could be effectively used to reduce experimental effort and time by reducing the number of experiments runs required to optimize the material attributes aimed at modeling the ECAP conditions for the following performance characteristics: impact toughness (I_T_), electrical conductivity (E_C_), hardness, and tensile characteristics of yield strength (σ_y_), ultimate tensile strength (σ_u_), and ductility (D_u_)

## 1. Introduction

Copper (Cu) and Cu alloys are highly preferred in a wide variety of industrial purposes, including the automotive, transportation, electronic, and electric sectors [1,2,3], as well as structural applications [4,5]. This is because of their superior conductivity, corrosion resistance [1,2,6], good formability, and significantly enhanced strength [4]. Furthermore, by severe plastic deformation (SPD) procedures, grain refinement reaching the ultrafine-grained (UFG) or nano-sized (NS) level creates better Cu with exceptional strength and ductility [7,8,9,10,11]. SPD approaches have the ability to enhance the mechanical, electrochemical, and physical characteristics of both pure and alloyed materials [12,13,14,15].

**Figure 1:**
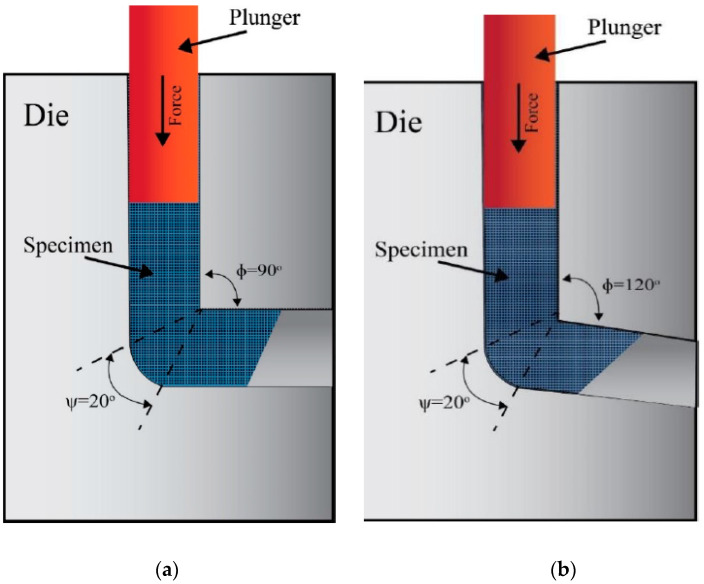
Schematic of the ECAP die with internal channel angle of (**a**) 90° and (**b**) 120°.

**Figure 2:**
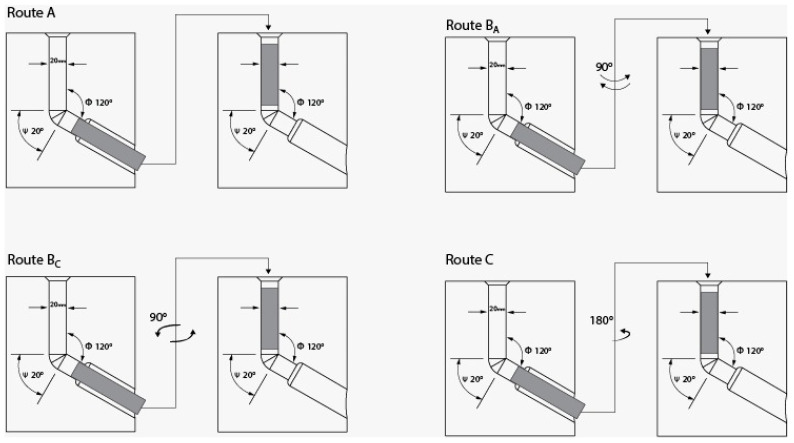
Schematic of the different routes of ECAP through multiple.

Various SPD methods are currently viable, including Equal-Channel Angular Pressing (ECAP) [16,17,18,19,20,21], high-pressure torsion (HPT) [22,23,24,25], accumulative rolling bonding (ARB) [26], twist extrusion (TE) [27], and Multi-channel spiral twist extrusion (MCSTE) [28,29,30,31]. ECAP is by far the most efficient SPD approach for generating UFG and nano-sized (NS) materials [32,33,34,35], along with enhancing ductility among the other SPD techniques [6,18]. ECAP is a viable choice for the advancement of various industrial applications due to its capacity to generate large-scale samples as opposed to SPD equivalents [36]. The ECAP die is made up of two channels with a similar cross-section that meet at a channel angle Φ and an angle of curvature Ψ (Figure 1). The shear strain produced on the processed materials is significantly affected by the channel die angle [6]. Furthermore, the ECAP processing route type has quite a considerable impact on both the microstructural development and mechanical characteristics of ECAPed billets [15], where the most prevalent ECAP route types A, Bc, and C as shown in Figure 2. While routes Bc and C rotate the processed sample by 90° and 180°, respectively, between succeeding passes, route A does not rotate the processed sample at all whereas at route BA the sample is rotated 90° in clockwise direction after the first pass and then rotated 90° in counter wise direction in the subsequent pass [15]. The following Equation (1) [17] can be utilized to compute the equivalent strain (*ε_eq_*) during ECAP processing in terms of the number of passes (N). As one of the SPD techniques, ECAP processing through multiple passes leads to imposing the sample to an equivalent strain greater than two, according to Equation (1).

(1) $$\varepsilon_{eq}=\frac{N}{\sqrt{3}}\left[2cot\left(\frac{\varphi +\psi }{2}\right)+\psi cosec\left(\frac{\varphi +\psi }{2}\right)\right]$$

Machine learning (ML) techniques have caught the attention of various academic and industry domains in recent years due to their exceptional performance in information retrieval from real-world data in today’s digital database era. As a result, ML technology has recently assumed a greater role in the development of novel nanomaterials [37,38,39,40]. ML techniques can handle complex material system functional changes fast, extract information from existing data, and anticipate characteristics [37,38,39,40]. Inference ML models operate as a data analysis tool, generating predicted outputs that are independent of the model constructed by accumulating training data from a given dataset, resulting in virtually realistic predictions with high accuracy. In this study, a predictive model was developed to estimate the ECAP parameters of Cu alloys using several ML algorithms, which included regression trees, Gaussian process regression, linear regression, support vector machine (SVM) for regression, ensemble regression trees, and regression artificial neural networks (ANN). These models were trained, validated, and tested using different evaluation metrics such as residuals, mean squared errors, root mean squared errors, and the determination coefficient (R^2^-score).

Few studies have examined ECAP performance in numerical terms. As a result, the current study seeks to determine the best ECAP conditions for the following performance characteristics: impact toughness (I_T_), electrical conductivity (E_C_), hardness, and tensile characteristics such as yield strength (σ_y_), ultimate tensile strength (σ_u_), and ductility (D_u_). Several experiments were carried out on pure commercial Cu with various ECAP settings, including the number of passes (N), ECAP die angle (φ), processing temperature, and route type.

## 2. Methodology

### 2.1. Experimental Procedure

Billets of commercially pure Cu (China Jingan Chemicals & Alloy Limited, Shanghai, China) were delivered as rolled rods 20 mm in diameter and 50 cm in length, with a chemical composition of weight% (0.5% Zn, 0.46% Si, traces of Sn, Mn, Al, and Fe, with the remaining being Cu). The ECAP billets, which have a 20 mm diameter and a 60 mm length, were formed by machining the as-received (AR) rods. After being annealed for two hours at 400 °C, the as-annealed (AA) copper billets were cooled in the furnace. Using two ECAP dies with internal channel angles of 90° and 120° and an outer corner angle ψ of = 20° as seen in Figure 1, the AA billets were processed via ECAP such as through as many as six passes of the various routes A, Bc, and C (Figure 2), resulting in equivalent strains of 1.054 and 0.634 per pass, respectively, in accordance with Equation (1). At a ram speed of 0.05 mm/s, ECAP processing was performed at three separate temperatures: room temperature (RT), 100 °C, and 200 °C. In order to minimize friction throughout the process, a graphite-based lubricant was utilized.

**Figure 3:**
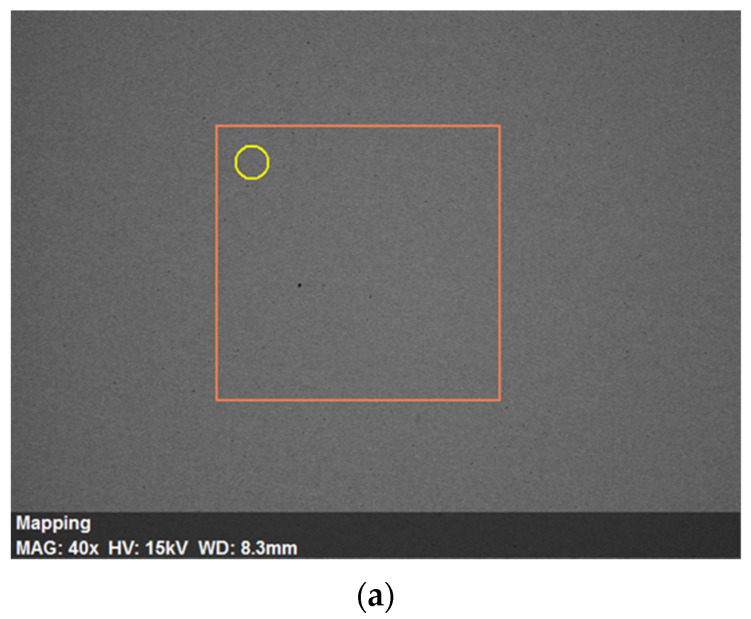

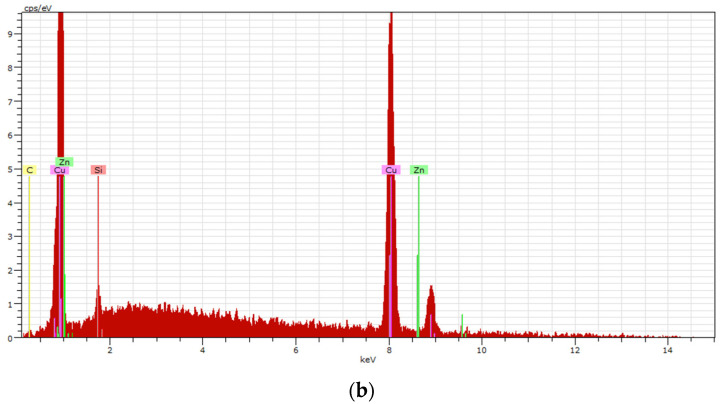
(**a**) SEM micrographs and (**b**) EDS analysis of the AR Cu billets (the orange frame shows the elemental mapping area, and the yellow circle shows the area of the EDS analysis).

The optical microscope (OM) (Qualitest, Nisku, Canada), and field emission scanning electron microscopy (SEM) (Joel Ltd., Tokyo, Japan) were used to examine the microstructure development of Cu billets. Cu billets were mounted, ground with 600, 800, 1000, and 1200 grit silicon carbide sandpaper, polished with alumina solution to a mirror-like sheen, and lastly, etched with a 3:1 volume ratio of HCl and HNO_3_ solutions. Energy-dispersive X-ray spectroscopy (EDS) (Joel Ltd., Tokyo, Japan) was used to analyze the chemical composition of the Cu billets. Figure 3 shows the SEM micrograph coupled with the energy-dispersive spectroscopy (EDS) analysis of the Cu billets.

A 100 kN universal testing machine (Instron 4210, Norwood, MA, USA) was used to conduct the tensile test on the Cu billets both before and following ECAP processing at RT and a strain rate of 10^−3^ s^−1^. The American Society for Testing of Materials’ (E8M/ASTM) guidelines for preparing tensile samples were followed in the preparation of the samples. Two tensile samples were machined from the middle of the Cu billets and evaluated individually for each processing condition. Furthermore, the AA and ECAPed Cu billets were sectioned across their center longitudinal axis perpendicular to the extrusion direction (ED), then ground and polished to a mirror-like surface. Vicker’s microhardness tests (HV) were performed for 15 s with a 1 kg applied force. The findings reported were the average of the recorded values for 5-equispaced indentations.

The Cu samples were tested for impact prior to and following the ECAP process utilizing Charpy V-notch test equipment (Time JB-W 500) (Shanghai Shenli Testing Machine Co., Ltd., Shanghai, China). The Cu samples are 55 mm long square bars and have a cross-section of 10 × 10 mm. A 45° angle, a radius of 0.025 mm, and a depth of 2 mm were used to produce the V-notch. Additionally, using an AG 4311B RLC-meter (Yokogawa Test & Measurement Corporation, Tokyo, Japan) at zero frequency, the electrical conductivity of the Cu billets was assessed at room temperature. For the Cu billets, a 20 mm diameter by 1 mm thick, thin circular disc was created. Four Cu wires were wrapped throughout the hole of a Teflon (or another non-conductive plastic block), bonded in place in a parallel manner with an exact spacing (gauge length) between the two inner wires. Prior to testing, the surfaces of the Cu billets were polished to remove any contaminants. The relative percentage of the international annealed copper standard (%IACS) was used to represent electrical conductivity.

### 2.2. Machine Learning Approach

The ECAP conditions of pure commercial Cu are the number of passes, die internal channel angle, ECAP processing temperature, and processing route. Three different levels were employed in this investigation, as shown in Table 1. In this study, the experimental design that was used to feed the machine learning techniques was based on a combination of ECAP’s conditions levels. Based on previous studies [41,42], 21 runs were performed and examined several ECAP responses, namely, Impact toughness (I_T_), Electrical conductivity (E_C_), Vicker’s microhardness (HV), and tensile characteristics such as yield strength (σ_y_), ultimate tensile strength (σ_u_) and ductility (D_u_) as shown in Table 2.

**Figure 4:**
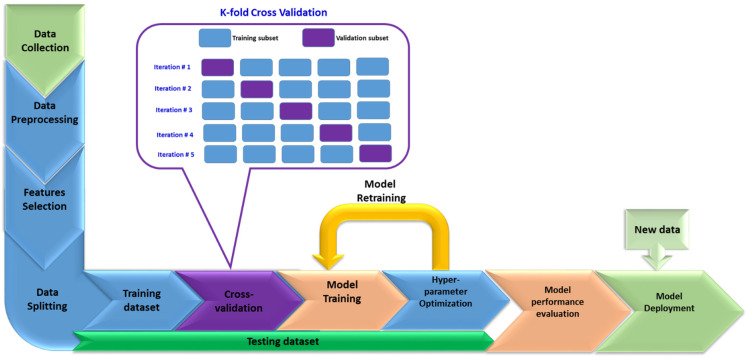
Workflow of ML modeling.

The workflow for developing ML models comprises several steps, as shown in Figure 4. Datasets were first gathered, then preprocessed, and then investigated. In order to assess the relative importance of each predictor in estimating the desired response, the correlation coefficients of all parameters were determined. The training set, the validation set, and the testing set were each given their own group of the dataset. Each dataset contributes significantly to the development, evaluation, and improvement of the inferential model. The training set is utilized for learning, and it accurately fits model variables. Most approaches for searching through training data for empirical relationships are data-appropriate in the sense that they can find associations that are obvious in training data but are not commonly done. A validation dataset, also known as the development set, is used to fine-tune the structure of variables from a workbook. The number of hidden layers, for instance, is the structure of the hyperparameter variables in ANN. The testing set is independent of the training set but should follow its same probability distribution, and the validation set should work as well. If a model that fits the training set also fits the test set, there is then a low degree of bias. Overfitting is frequently indicated by a better fit of training sets other than the test set. As a result, a test set is solely utilized to evaluate the model’s performance and directly examine its generalization and inference to previously unseen data. In order to avoid overfitting, the cross-validation (CV) methodology is employed in the modeling strategy of this work. The k-fold CV technique divides the training set into randomly selected small subsets known as folds. The model is trained using the folds as training data for each k-fold, and the resulting model is verified using the remaining data as a test set to compute the model performance measure. This step is repeated k-times in a loop, and the model performance measure is then calculated by averaging the results obtained in the loop. This technique is expensive, but it saves data, especially when the model is trained on a small set of data. After successful modeling training and testing, it can be deployed to infer new unseen input data to predict the targeted parameter or material property under investigation.

Similar to other fuzzy and ANN approaches [43,44], the techniques used in this study to estimate the ECAP parameters of Cu alloys include regression artificial neural networks, support vector machine (SVM) for regression (SVR), ensemble regression trees, linear regression. These techniques are briefly explored in the sections that follow.

#### 2.2.1. Linear Regression

In general, the relationship between one or more predictor (input) variables and a response (output) variable is often defined using linear regression models. By using supervised machine learning, the linear regression method finds the best-fitting linear relationship between the predictor(s) and the response output variable. It helps with the comprehension and forecasting of complex system behaviors as well as the analysis of experimental, financial, and biological data. The output response vector *Y* for multiple input variable (*X*) linear regression is given by:

(2) $$Y=\beta_{0}+\sum_{n=1}^{N}\beta_{n}X_{n}+\epsilon_{n}$$

where *β_n_* represents the estimated linear parameters, *β*_0_ is constant, and *ϵ_n_* signifies the error terms.

#### 2.2.2. Regression Trees

Regression trees are decision trees using continuous values rather than class labels as the target variables in the leaves (decision branches), where nodes, branches, and leaves are used to divide data into subgroups aiming at forecasting the output response. The regression trees technique employs enhanced split decisions and appropriate halting rules, and it may be used to effectively express decisions, locate probable occurrences, and identify potential repercussions.

#### 2.2.3. Ensembles of Regression Trees

An ensemble of trees is a technique that employs a set of rules created from continuous patterns found in each dataset to provide judgments and final values of predictions. The primary premise of the technique is to categorize data from the training dataset into a binary tree structure with nodes using a recursive methodology. At each step, the cases in the current node, known as the parent node, can be divided into two child nodes based on the value of a predictor variable. The average of the response values in a terminal node serves as its expected value. After the tree has been built, any branches that do not advance the model are removed, leaving a final trimmed tree.

#### 2.2.4. Regression Gaussian Process

The GPR regression approach is a nonparametric Bayesian regression strategy that has recently achieved major advances in machine learning. The approach has various advantages, including the ability to perform well on small datasets and offer uncertainty estimates on predictions.

#### 2.2.5. Support Vector Machine

In the ML modeling of classification or regression problems, the SVM technique [45] is frequently utilized. The support vector machine regression (SVR) employs equivalent classification techniques as the SVM with minor modifications [45,46,47,48,49]. A margin of tolerance (*ε*) is given as an expected prediction to the SVM that the problem would previously be requested throughout the regression case; furthermore, there is a more challenging explanation to be considered, which is that the algorithm is more complex. The SVM approach divides data into several classes with the highest margin by choosing the best hyperplane. The training dataset (*x_i_*, *y_i_*), with *I* = 1, 2, …, *N*, is best approximated using the function *f*(*x*), where *x_n_* is a multivariate collection vector or matrix. The function *f*(*x*) may be expressed in its simplest form as [45]:

(3) f(x)=wx+b

The ideal values of *w* and *b* are obtained by minimizing the following formula [45]:

(4) $$\min\;\frac{1}{2}{\left\|W\right\|}^{2}+C\sum_{i=1}^{N}\left(\xi_{i}+\xi_{i}^{*}\right)$$

Subject to:

(5) $$\{\begin{array}{c} y_{i}-wx_{i}-b\leq \varepsilon +\xi_{i}\\ wx_{i}+b-y_{i}\leq \varepsilon +\xi_{i}^{*}\\ \xi_{i},\xi_{i}^{*}\geq 0 \end{array}$$

where the ε-insensitive tube expresses the error tolerance and C is a balance between the empirically estimated error and the generic term. The regression function could well be written as follows, utilizing LaGrange multipliers from the optimal constraint’s algorithm [45]:

(6) $$y=\sum_{i=1}^{N}\left(a_{i}+a_{i}^{*}\right)K\left(x_{i},x\right)+b$$

The kernel function is denoted by *K*(*x_i_*, *x*). It is worth mentioning that the most well-known kernel functions are the linear, polynomial, sigmoidal, Gaussian, and radial basis functions. The latter function is represented by the following formula [45]:

(7) $$K\left(x_{i},x_{j}\right)=\exp\left(-\frac{{\left\|x_{i}-x_{j}\right\|}^{2}}{2\sigma^{2}}\right)$$

where *σ* is the spread of the kernel function’s distribution and $\left\|x_{i}-x_{j}\right\|$ is the Euclidean distance that separates the two feature vectors.

#### 2.2.6. Artificial Neural Network

**Figure 5:**
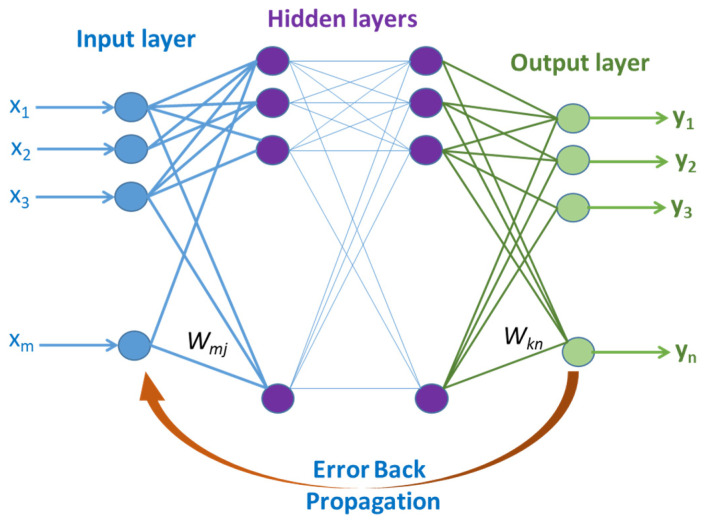
The generic architecture of ANN.

ANN is a deep learning algorithm that simulates how neurons in the human brain operate. Convolutional neural networks, recurrent neural networks, and vanilla neural networks are a few of the several forms of ANN. Recurrent neural networks and convolutional neural networks are both very good at handling unstructured data, whereas vanilla neural networks can only handle structured data. The input, hidden, and output layers are the three principal kinds of layers that make up an ANN’s architecture. As seen in Figure 5, each level contains a functional node, namely a neuron. Each neuron in each layer is to be paired with an activation function. For regression ANN, the output (*y*) is only one node that is collected from any previously activated node (*j*) and is computed by the activation function *f* as follows:

(8) $$y=f\left(\sum_{i=1}^{m}\left(W_{ij}x_{i}+b_{j}\right)\right)$$

where *W_ij_* is the weight of the path between any two nodes (*i*, *j*) in a network layer, *x_i_* is the input to the layers, and *b_j_* is the bias term added to the node. Numerous activation functions, including sigmoid, softmax, tanh, rectified linear unit (RelU), leaky RelU, and others, are frequently utilized with ANN architecture. The activation function is in charge of introducing nonlinear activity into the network layers flow. In order to prevent the overfitting issue; regularization terms are therefore coupled with each layer. The weighted-based computation of errors in either the forward or back direction is the link between different layers. In forward propagation, the output is calculated by multiplying and adding the weights of each feature. Then the result is therefore given a biased term. Backward propagation is the process of adjusting model weights depending on the difference between actual and estimated output errors. This method requires the use of an optimization process to minimize what is called the loss function.

The model’s performance and generalization behavior have been assessed after training it and refining its hyperparameters. Different evaluation measures, such as residuals, the determination coefficient (R^2^-score), mean squared errors (MSE), and root mean squared errors (RMSE), were utilized for this purpose.

## 3. Results

### 3.1. Microstructural Evolution

**Figure 6:**
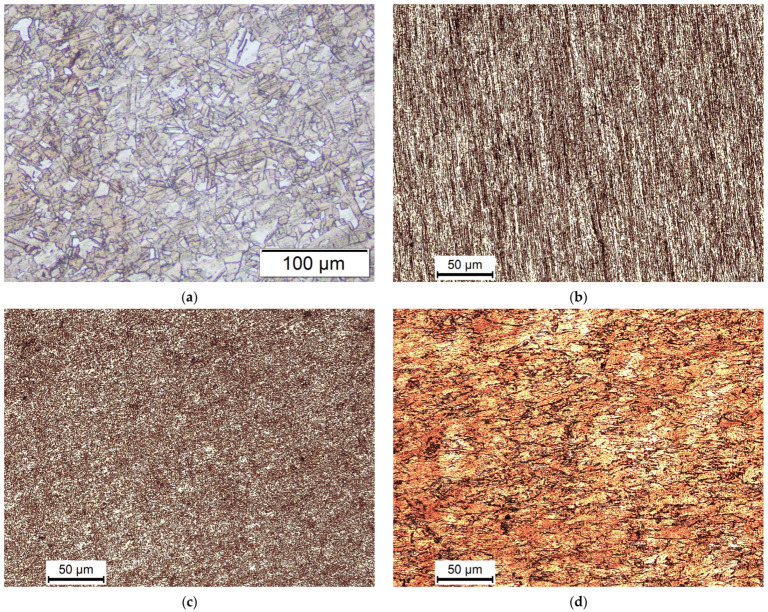

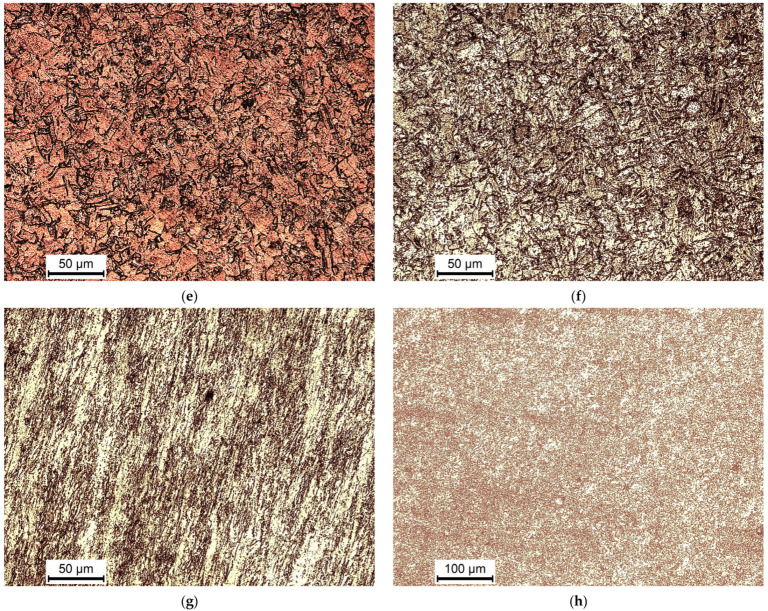
OM micrographs of the AA (**a**) Cu billets and ECAPEed billets processed through 2-Bc _90°die (**b**), 6-Bc_90°die (**c**), 6-A_90°die (**d**), 6-C_90°die (**e**), 6-Bc_90°die (**f**) 2-Bc_120°die (**g**) and 6-Bc_120°die (**h**) at RT (**b**–**e**,**g**,**h**) and at 200 °C (**f**).

An OM was used to analyze the Cu samples subjected to the seven distinct parameter sets along with the AA sample in order to evaluate the microstructural development; Figure 6 displays their micrographs. Figure 6a shows that the AA sample was largely made up of irregular coarse grains. However, the grain sizes varied from extremely fine to very coarse. The samples processed by 2-Bc at φ = 90° & RT, shown in Figure 6b, were examined, and it was found that processing produced a structure that seemed to be a UFG that was oriented parallel to the ED. Figure 6c shows how processing with 4-Bc at RT resulted in exceptional grain refinement. The subsequent sample revealed a totally recrystallized microstructure with UFG equiaxed grains as its dominant component.

Cu billets were processed via six passes of both route A and route C (6-A and 6-C, respectively) utilizing the 90°- die at RT to assess the impact of the ECAP route type on the microstructure. Processing via 6-A showed a substantial UFG structure, as illustrated in Figure 6d, corresponding to the 6-Bc situation. On the contrary, it was discovered that route A produced more elongated grains than route Bc produced equiaxed grains, which matched with the earlier study [50]. In contrast to route Bc, processing through 6-C resulted in grains that were more coarsely equiaxed (Figure 6e). It is important to note that following route C processing even passes, the plastic strain from the earlier odd passes is reverted, leaving the equiaxed grains intact, which was consistent with previous research [51]. In addition, the Cu billet was processed via 6-Bc utilizing a 90-degree die at 200 °C to assess the processing temperature effect on the microstructure of the ECAP processed Cu, as presented in Figure 6f, where raising the processing temperature up to 200 °C had a substantial influence on grain size, with coarser equiaxed grains dominating the microstructure compared to the RT processed equivalents.

The effect of the ECAP dies angle on the Cu microstructure was studied by examining the OM micrographs of two separate sets of billets. Both sets were processed at RT with 2-Bc and 6-Bc; one set was processed with φ = 90° (Figure 6b,c) and the other with φ = 120° (Figure 6g,h). The microstructures of the billets that underwent 90° and 120° 2-Bc processing both exhibited parallel EDs and UFGs, as illustrated in Figure 6b,g. The reduced plastic strain coupled with the 120° die angle, as shown by Equation (1), results in grains with lower shear orientation angles than their 90° 2-Bc counterparts, as seen in Figure 6g. However, processing with the 90° die created finer grains. Lastly, 6-Bc processing produced UFG structures employing both die angles.

### 3.2. Analysis of Machine Leaning Approach for Mechanical and Electrical Behaviour

**Figure 7:**
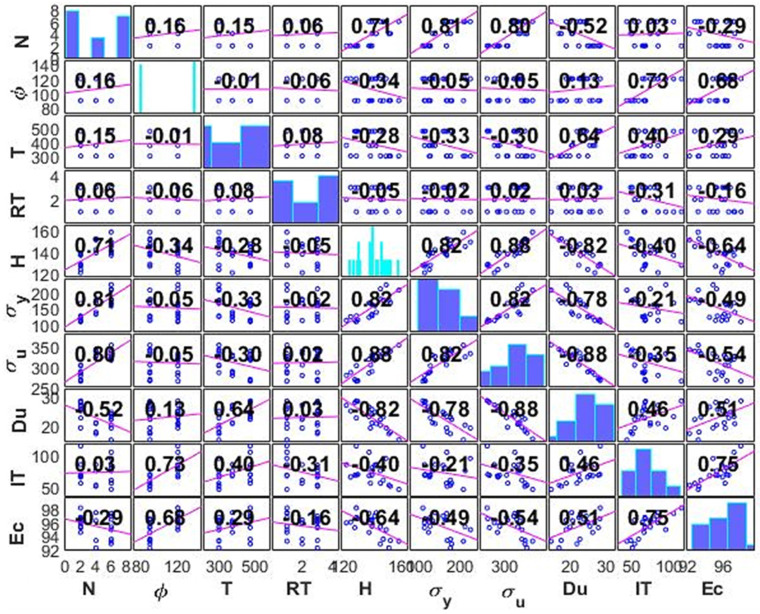
Correlation plots of all response ECAP parameters (HV, σ_y_, σ_u_, D_u_, I_T_, and E_C_) versus Alloy feature parameters (N, ϕ, T, and RT).

The correlation graph for the input and output variables is shown in Figure 7. The correlation coefficients for each pair of variables in the input data matrix are displayed. A two-variable scatterplot with a least-squares reference line whose slope is equal to the reported correlation coefficient is used for each off-diagonal subplot. Each diagonal subplot shows a histogram of each variable’s distribution. The estimated Pearson’s coefficient, a measure for determining the linear correlation between two sets of data, provides the foundation for this figure. The correlation coefficient always ranges from −1 to 1 in value. The positive values for the two variables in this diagonally symmetrical chart imply an upward slope, whilst the negative values suggest a downward slope. A high correlation is often implied by coefficients with values of one or near to one, whilst a weak correlation is generally implied by coefficients with values close to zero. This correlation study is helpful to initially determine the weights of each input feature and its importance in building the predictive model. The bar charts represent the data distribution of each variable of the chart; this is instead of displaying the correlation coefficient for each variable with itself, which is always 1.

It can also be seen from the correlation plot that increasing the number of passes (N) has positively affected the hardness and the tensile properties of yield strength (σ_y_) and ultimate tensile strength (σ_u_), where a high positive significant correlation is reached at 0.81 and 0.8, respectively, whereas D_u_ showed a negative correlation at −0.52. These outcomes are supported by the experimental findings where the ECAPed billets showed a considerable rise in σ_y_ and σ_u_ compared to AA equivalents. It should be noted that with the increase in strength, the ductility decreases consequently. It is also revealed that increasing the ECAP die angle (φ) has a positive effect on E_C_ and I_T_, with a strong positive significant correlation at 0.68 and 0.73, respectively. In the case of I_T_, this may be attributed to the lower strain that was applied. Moreover, it can be seen that increasing the processing temperature has positively affected the D_u_, where a high positive, strong correlation is reached at 0.64, where the temperature increase led to an increase in grain size leading to coarse grains.

### 3.3. Effect of ECAP Processing Parameters on Cu Properties

#### 3.3.1. Hardness Distribution

**Figure 8:**
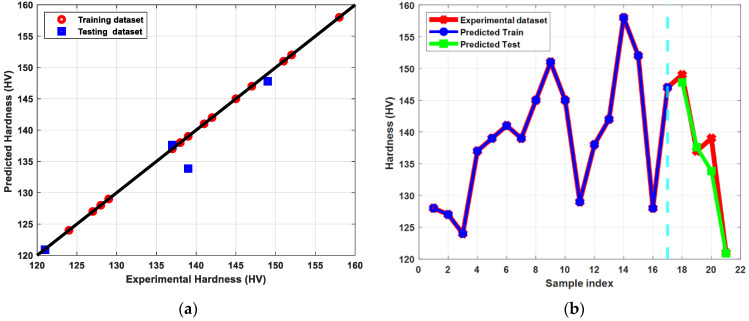
(**a**) Predicted versus experimental hardness evaluated for the training and the testing sets, (**b**) Predicted hardness versus sample index plotted for predicted train, predicted test, and experimental hardness.

The ECAP parameters of Cu were modeled using different ML algorithms, including regression trees, ensembles of three, linear regression, support vector regression, Gaussian process regression, and artificial neural networks, as previously mentioned. The training and testing datasets were randomly selected from the experimental data to be 80% and 20% of the input for the training and testing, respectively. Both sets possess the same statistical distributions as the original (experimental) data. RMSE and R^2^-score were used to assess the model’s performance after training. The training set’s best RMSE and R^2^ values for the hardness parameter were 0.63 and 0.99, respectively; on the other hand, these figures were 2.65 and 0.93, respectively, for the testing set. According to Figure 8, both the training and testing sets of hardness showed that the prediction values were extremely close to the actual data provided to the model. These findings were obtained by optimizing an ANN with input, output, and three hidden layers, as well as using rectified linear unit (ReLU) activation functions. According to Table 3, the other algorithms also performed well throughout both training and testing; however, the carefully tuned ANN described earlier outperformed the other chosen algorithms. The use of LR, SVR, and ANN met the criteria for a model with accepted accuracies that should achieve satisfying scores for both the training and testing set.

The AA billets displayed almost constant hardness values across both the longitudinal and transverse sections with an HV average value of 100. According to the previous results, ECAP processing resulted in increasing the HV- values of the Cu billets. Moreover, several passes increased the uniformity of the hardness distribution since both the center and peripheral portions reported overly near hardness values. Furthermore, the build-up of plastic strain during many passes increased the hardness values in both the core and peripheral areas, which matches the findings of Djavanroodi et al. [52]. The ECAP procedure changed a high density of low-angle grain boundaries (LAGBs) into high-angle grain boundaries, resulting in the UFG structure shown in Figure 6. The preceding took place as a result of dislocation creation, multiplication, and motion throughout ECAP processing [6,14,16,17], resulting in dislocation motion impedance and, as a result, a stronger Cu billet [14,16,41,42]. As a result, it is possible to conclude that grain refinement is an efficient strengthening process that leads to increased Cu hardness [6,41,42].

Route type is an additional significant factor that influenced the features of the processed billets; it has been established that route type Bc provides the highest efficient route for refining grains and produces an almost equiaxed UFG structure, as shown in Figure 6c. As a result, route Bc, which has the most grain refinement among other types, is the most successful route in enhancing the hardness of ECAPed billets. Contrarily, route A produced finer grains that were more elongated (Figure 6d), whilst route C produced coarser grains that were nearly equiaxed, in comparison to the other route types as shown relative to the other route types as shown in Figure 6e. In comparison to the other route types, route A produced more elongated fine grains (Figure 6d), but route C produced almost equiaxed coarser grains (Figure 6e). As a result, when compared to counterparts processed via route Bc, the sample processed via route A showed lower hardness values in both the central and peripheral areas; however, when opposed to counterparts processed via route C, it recorded greater hardness values in both the central and peripheral regions.

In addition, although the sample processed at 100 °C and 200 °C indicated higher hardness values in the center and near the periphery, ECAP processing at higher temperatures demonstrated a more homogeneous distribution of the hardness, which was consistent with the prior work [6].

Previous research has shown similar results. Xu et al. [11] found that processing pure Cu via 8-Bc at RT increased the hardness by 45.3%. According to Dalan et al. [53], RT processing was more successful in enhancing hardness owing to grain refining than ECAP processing at 300 °C. While Jayakumar et al. [12] claimed that the Cu-Cr-Zr alloy’s hardness reached saturation after 4-Bc employing a 105°-die at RT and that increasing the number of passes beyond 4-passes exhibited a negligible difference. According to Wei et al. [54], ECAP processing using 4-Bc increased the Vicker hardness of Cu billets from 63 to 145. Moreover, when Cu-0.1 wt% Zr was processed by ECAP through 8-Bc at RT, the Vicker’s microhardness increased from 95 to 143 HV. Ngam et al. [55] ascribed this response to the influence of refining the grain size on increasing the strength of the alloy in accordance with Hall-Petch [15,56]. According to Tong et al. [57], the Cu-0.36Cr-0.49Zr alloy’s Vicker’s microhardness dramatically rose during the first ECAP processing before remaining almost constant over the subsequent eight runs of processing. Tong et al. [57] showed a comparable response for Cu-0.25Se-0.25Te Alloy as Huang et al. [9].

#### 3.3.2. Tensile Properties

**Figure 9:**
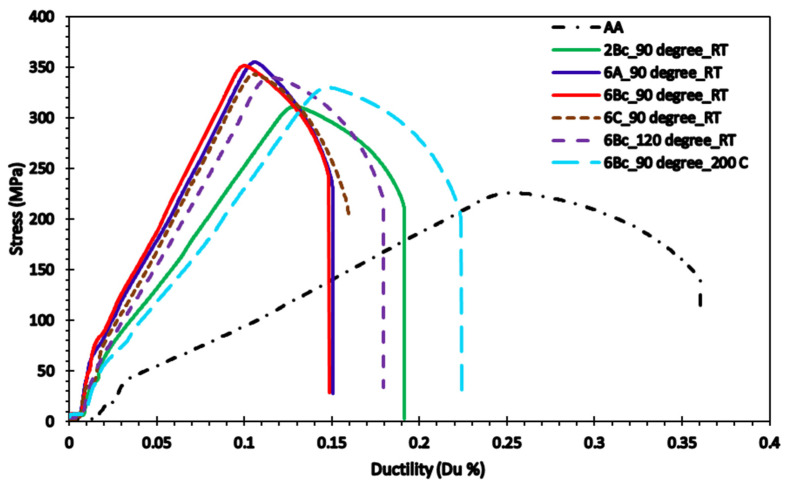
Stress-strain curves of Cu billets before and after ECAP processing.

**Figure 10:**
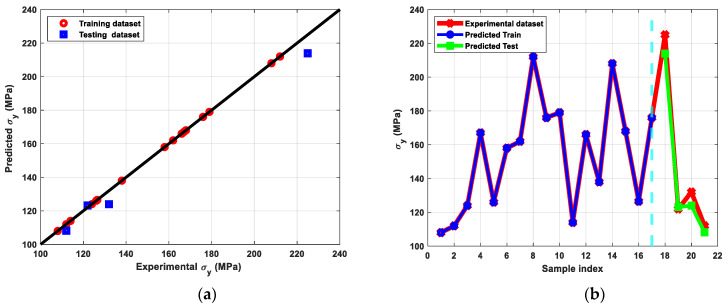
(**a**) Predicted versus experimental σ_y_ evaluated for the training and the testing sets, (**b**) Predicted σ_y_ versus sample index plotted for predicted train, predicted test, and experimental σ_y_ datasets.

**Figure 11:**
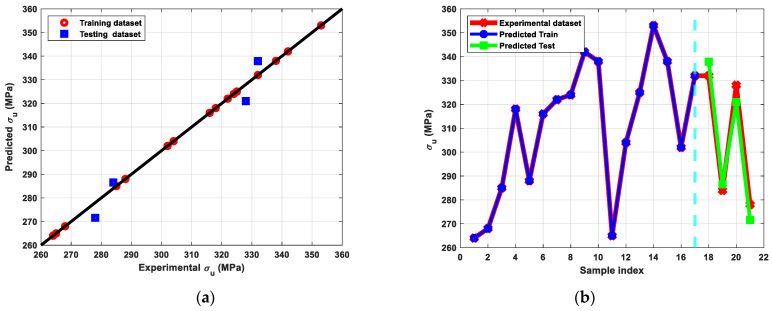
(**a**) Predicted versus experimental σ_u_ evaluated for the training and the testing sets, (**b**) Predicted σ_y_ versus sample index plotted for predicted train, predicted test, and experimental σ_u_ data.

**Figure 12:**
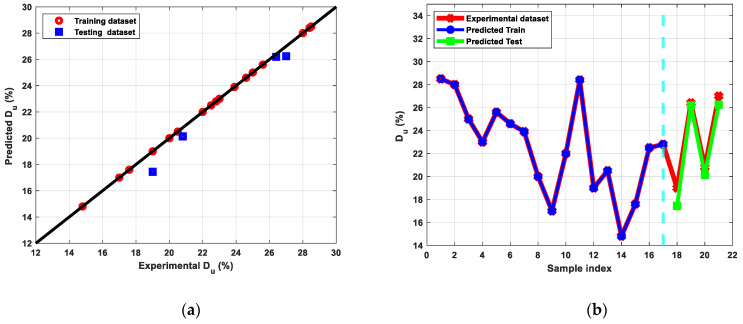
(**a**) Predicted versus experimental D_u_ evaluated for the training and the testing sets, (**b**) Predicted Du versus sample index plotted for predicted train, predicted test, and experimental D_u_.

Figure 9 shows the stress-strain diagram of the Cu billets before and after ECAP processing through different parameters. In addition, Figure 10, Figure 11 and Figure 12 show the results of modeling Cu’s tensile characteristics, including σ_y_, σ_u_, and D_u_. The results showed a high correlation between the actual dataset received from the experiments and the predicted dataset. Nearly all of the used algorithms performed well throughout data training and testing, but the tuned ANN with three hidden layers and the RelU activation function produced the best outcomes, as evidenced by the RMSE and R^2^-scores shown in Table 4.

The AA Cu billets displayed σ_y_, σ_u_, and D_u_ of 82 MPa, 225 MPa, and 36%, respectively. Tensile characteristics showed that ECAPed billets have a considerable rise in σ_y_ and σ_u_ combined with no notable compromise in ductility when compared to AA equivalents. Additionally, Figure 10 showed that the best σ_y_ was produced under the processing settings 6-Bc, 90°-die, and 298 °C, indicating that route Bc is the most efficient route for raising the σ_y_ owing to the large reduction in grain size (Figure 6c). Moreover, according to Equation (1), the increased impost plastic strain from the 90°-die led to a considerable improvement in the σ_y_. Furthermore, processing in the RT led to a considerably reduced grain size and hence improved σ_y_. Also, the increase in dislocation density, which limits the mobility of the dislocation, led to the buildup of shear strain up to six passes [54]. However, utilizing the 90°-die at RT, both routes A and Bc resulted in values too near to the maximum of σ_u_ at 353.4 and 351.4 MPa, respectively.

The drop in Cu D_u_ following ECAP processing could be ascribed to grain size refining. Furthermore, Equation (1) demonstrated that the 120°-die experienced better D_u_ than the sample processed via the 90°-die because of the reduced applied strain of the 120°-die. Additionally, the processing temperature has a big impact on how ductile the copper is since strain hardening occurred during RT processing, which is why lesser ductility was observed. On the other side, raising the ECAP processing temperature led to strain softening and, thus, greater ductility. Moreover, it was obvious that route Bc is the most successful route in grain refining. Therefore, it demonstrated reduced ductility when compared to the other route types.

Previous research has shown identical trends. Wu et al. [58] showed a considerable drop in the D_u_ up to 22% and a large rise in the σ_u_ and σ_y_ of pure Cu billets following ECAP processing via route Bc at RT, which they ascribed to the grain refinement strengthening. According to Wei et al. [54], 4-A processing at RT increased the σ_u_ and σ_y_ of pure Cu from 62 and 121 MPa to 365 and 389 MPa, respectively, while decreasing the D_u_ from 50.1 to 19.8. While Wang et al. [59] showed that 12-Bc at RT increased the σ_u_ and σ_y_ of pure Cu from 89.6 and 222 MPa up to 403.9 and 440.8 MPa, respectively, while significantly reducing the D_u_ up to 14.5. Tian et al. [60] used a 90-°die at RT to study the influence of ECAP route type on the tensile characteristics of a Cu-8wt.% Ag alloy. When compared to routes Bc and C, they discovered that 4-A was more successful in raising both the σ_u_ and σ_y_, as well as elongation at fracture. ECAP processing was employed by C. Ngam et al. [55] for 8-Bc at RT to raise the σ_u_, σ_y_, and du as well as to compare to the AA equivalent. Zhu et al. [61] identified a common trend for Cu-0.2wt% Mg and Cu-0.4wt% Mg alloys, as did Ko et al. [62] for Cu-3 wt% Ag alloy and Ni et al. [63] for Cu-1 wt% TiC alloy.

#### 3.3.3. Impact Toughness

**Figure 13:**
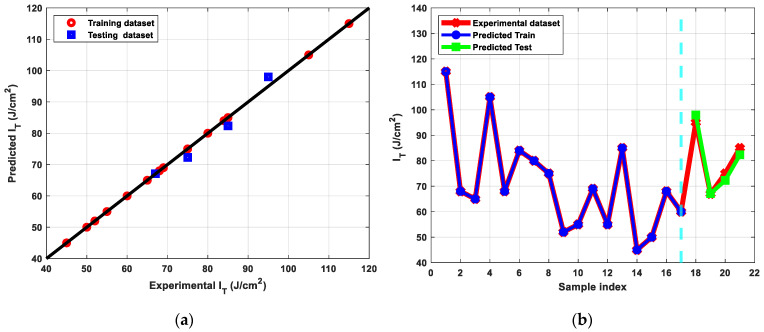
(**a**) Predicted versus experimental I_T_ evaluated for the training and the testing sets, (**b**) Predicted I_T_ versus sample index plotted for predicted train, predicted test, and experimental I_T_.

The results of the ML modeling of the I_T_ parameter are shown in Figure 13, where the predicted outcomes for both the training and testing phases are highly correlated to the actual experiment dataset. Once more, the best outcomes produced by ANN outperform those produced by the other algorithms, as displayed in Table 5. The R^2^ metric’s negative and small values showed that algorithms other than ANN were unable to extract useful information from the input data, whereas ANN with tuned hyperparameters demonstrated high prediction accuracy.

The AA Cu billets displayed an impact toughness of 60 J/cm^2^. With the exception of a few ECAP samples that were processed at RT, the bulk of the ECAPed billets showed increased absorbed impact energy compared to the AA (Table 5), which was consistent with other findings [64,65]. In light of this, it is significant to highlight that processing ECAP at higher temperatures improved the amount of impact energy that was absorbed. Contrarily, the Cu sample processed via 6-Bc utilizing the 90-degree die at 200 °C displayed lesser impact energy than the AA, which might be explained by the existence of brittle particles at the grain boundaries in accordance with the earlier work [65]. Additionally, the 120°-die had a larger impact energy than the 90°-die, which may be ascribed to the lesser strain that was applied, according to the impact data (Table 5). According to Fang et al. [65], ECAP processing caused metals to shift from brittle to ductile mode after several passes, which is associated with the transition of the microstructure due to the high sensitivity of impact energy to microstructural changes [56]. They also said that ECAP processing, despite the fact that it reduced the elongation of the ECAPed samples, might result in tougher alloys [65,66]. According to Jiang et al. [66], the impact energy of Al-26 wt.%Si improves significantly after grain refining. They said that the alloy’s maximum absorbed energy was discovered to be close to RT and that this temperature of peak impact energy rises with the number of ECAP processing passes. Additionally, Meyer et al. [67] showed an increase in impact energy of magnesium alloy AZ31B following ECAP processing at 260 °C for up to four passes.

#### 3.3.4. Electrical Conductivity

**Figure 14:**
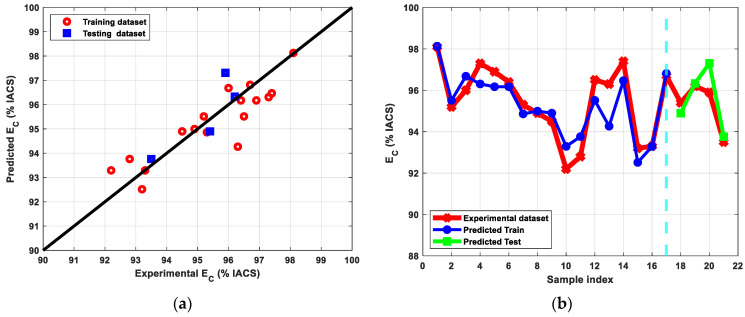
(**a**) Predicted versus experimental E_C_ evaluated for the training and the testing sets, (**b**) Predicted E_C_ versus sample index plotted for predicted train, predicted test, and experimental E_C_.

The results of the modeling of Cu’s electrical conductivity are shown in Figure 14. The ANN was able to accurately capture the model’s trend whereas other algorithms were unable to do so. According to Table 6, the optimized network obtained R^2^ values of 0.81 and 0.97 for the training and testing sets, respectively. As a result, this model inference is appropriate for predicting the E_C_ for new unseen input data under various experimental setting combinations.

The E_C_ of the AA billet was 99.4% IACS, although processing produced minor declines in E_C_ values, ranging from 92.2% to 98.1% IACS, as indicated in Table 6. The aforementioned findings give clear proof that ECAP processing may be utilized to reinforce Cu billets without affecting their E_C_, which is consistent with previous findings [68]. This is demonstrated by the notion that processing for up to 6-Bc led to a maximum reduction of 6.6% in the E_C_ compared to the AA samples and that the E_C_ decreased with the number of passes. The increase in the number of dislocations, grain boundaries, and other defects generated by ECAP processing resulted in electron scattering [5]. Identical behaviors, as well as increases in E_C_ with processing temperature, were observed by Zhao et al. for the Al-Mg-Si alloy [69]. They also discovered that increasing diffusion kinetics could result in the breakdown of impurities, however, only for the Cu-0.81Cr-0.07Zr alloy, which was supported by Dalan et al. [53,70]. Furthermore, Dalan et al. came to the conclusion that the E_C_ was unaffected by the number of ECAP passes. They also determined that route Bc delivered greater E_C_ than route A for a sample processed for four passes. According to the research of Zhu et al. [61], the E_C_ of the Cu-Mg alloy decreases with increased grain refinement, as well as with an increase in the density of dislocations and point defects, which results from intensive processing. According to Kumar et al.’s research’s [71], the E_C_ of oxygen-free, highly conductive Cu is influenced by the defect density created by cyclic channel die compression and decreases as defect density increases. Research on the Cu-3 wt% Ag alloy and pure Cu by Ko et al. [62] and Wei et al. [54] further verified the previously documented drop in EC with the number of ECAP passes. However, Ciemiorek et al. [72] discovered that after the first pass, the E_C_ of Cu dropped, only for the E_C_ to climb once again with the addition of subsequent passes until there were eight. Finally, Cho et al. [73] studied the Cu-15Ag alloy and discovered that for 8-pass processing, the greatest E_C_ values were obtained, in decreasing order, by routes C, A, and Bc.

## 4. Conclusions

In this paper, ML approaches of linear regression, regression trees, ensembles of regression trees, Gaussian process, support vector regression, and artificial neural networks was used in the modeling training and testing stages to imitate the influence of ECAP processing parameters on the mechanical and electrical characteristics of pure Cu, including the number of passes (N), ECAP die angle (φ), processing temperature, and route type. Several experiments were conducted on pure commercial Cu while altering the ECAP processing parameters settings. Model predictive ability was assessed using accurate measures such as root-mean-squared errors and R^2^-scores. The approaches presented here may be utilized to successfully reduce the amount of effort and time spent on experimental work by reducing the number of experiments runs required to optimize the material properties aimed at modeling the ECAP conditions for the following performance characteristics: impact toughness (I_T_), electrical conductivity (E_C_), hardness, and tensile characteristics of yield strength (σ_y_), ultimate tensile strength (σ_u_), and ductility (D_u_). The following conclusions could be drawn:

1. The 90°-die showed to be more effective in producing a UFG grain structure compared to the 120°-die.
2. Route Bc is the most efficient route type in grain refining, resulting in the highest hardness of ECAPed billets.
3. ECAP processing at higher temperatures demonstrated a more homogeneous distribution of the hardness.
4. 6-Bc processing through the 90°_die at RT resulted in increasing the HV by 72% compared to the AA condition.
5. The carefully tuned ANN for hardness outperformed other adopted ML algorithms throughout both training and testing sets with the lowest RMSE and highest R^2^ values, showing that the prediction values were extremely close to the actual data provided to the model.
6. ECAPed billets showed a considerable rise in σ_y_ and σ_u_ combined with no notable compromise in ductility when compared to AA equivalents.
7. Route Bc demonstrated the most reduced ductility and the highest σ_y_ when compared to the other route types.
8. Processing at 200 °C resulted in increasing the ductility of the ECAPed billets compared to the counterparts processed at RT.
9. 6-A processing through the 90°_die at RT resulted in increasing the ultimate tensile strength by 56% compared to the AA condition.
10. The ECAP die with a channel angle of 90° is more effective in increasing the tensile strength of the Cu billets.
11. The tuned ANN for tensile properties with three hidden layers and the RelU activation function generated the best results among other algorithms, as indicated by the RMSE and R^2^-scores, with a strong correlation between the actual dataset from the experiments and the predicted dataset.
12. The majority of the ECAPed billets showed increased absorbed impact energy compared to the AA.
13. Processing ECAP at higher temperatures improved the amount of impact energy that was absorbed.
14. The Cu sample processed via 6-Bc utilizing the 90-degree die at 200 °C displayed lesser impact energy than the AA.
15. The 120°-die had a larger impact energy than the 90°-die, which may be ascribed to the lesser strain that was applied.
16. The ANN generated the best results for impact toughness, surpassing the other algorithms, which were unable to extract valuable information from the input data, while ANN with tuned hyperparameters demonstrated high prediction accuracy.
17. ECAP processing can strengthen the Cu billets without a significant effect on their electric conductivity.
18. The ANN was able to accurately capture the model’s trend for EC with low RMSE and high R^2^ values, whereas other algorithms were unable to do so, showing that ANN model inference is appropriate for predicting the EC for new unseen input data under various experimental setting combinations.

## Figures and Tables

**Table 1:** ECAP parameters and corresponding levels.

ECAP Conditions	Symbol	Unit	Conditions Levels		
			−1	0	1
Number of passes	N	Pass	2	4	6
ECAP die angle	φ	°	90	120	-
Processing temperature	T	K	298	373	473
Processing route type	-	-	A	Bc	C

**Table 2:** Design of experiment of ECAP parameters and process response.

Run	ECAP Condition				ECAP Response					
	N	φ	T	Processing Route Type	Hardness	Tensile Strength			I_T_ (J/cm^2^)	E_C_ (% IACS)
					(HV)	σ_y_ (MPa)	σ_u_ (MPa)	Du (%)		
AA	0				100	82	225	36	60	99.4%
1	2	120	473	A	128	108	264	28.5	115	98.1%
2	2	90	473	C	127	112	268	28	68	95.2%
3	2	120	298	C	124	124	285	25	65	96%
4	6	120	473	A	137	167	318	23	105	97.3%
5	2	90	373	A	139	126	288	25.6	68	95.4%
6	6	120	473	C	141	158	316	24.6	84	96.9%
7	6	120	473	C	139	162	322	23.9	80	96.4%
8	6	120	298	C	145	212	324	20	75	95.3%
9	6	120	298	A	149	225	332	19	95	94.9%
10	2	90	373	A	137	122	284	26.4	67	94.5%
11	4	90	298	C	151	176	342	17	52	92.2%
12	4	120	373	C	139	132	328	20.8	75	96.2%
13	6	90	473	Bc	145	179	338	22	55	92.8%
14	2	90	473	C	129	114	265	28.4	69	96.5%
15	2	90	298	Bc	138	166	304	19	55	96.3%
16	4	120	373	A	142	138	325	20.5	85	97.4%
17	6	90	298	A	158	208	353	14.8	45	93.2%
18	4	90	298	C	152	168	338	17.6	50	93.3%
19	2	120	298	A	128	126.5	302	22.5	68	96.7%
20	2	120	373	Bc	121	112	278	27	85	95.9%
21	6	90	473	Bc	147	176	332	22.8	60	93.5%

**Table 3:** Evaluation metrics of Hardness machine learning models for both the training and testing sets.

	Training Set		Testing Set	
ML Algorithm	RMSE (HV)	R^2^	RMSE (HV)	R^2^
Linear regression	3.29	0.87	3.77	0.86
Regression trees	4.44	0.78	7.11	0.49
Ensemble of trees	0.63	0.99	6.82	0.54
Gaussian process regression	0.62	0.99	10.45	0.08
Fine Gaussian SVR	3.36	0.87	3.46	0.88
Artificial neural networks	0.63	0.99	2.65	0.93

**Table 4:** Evaluation metrics of σ_y_ machine learning models for both the training and testing sets.

Parameter		Training Set		Testing Set	
	ML Algorithm	RMSE (MPa)	R^2^	RMSE (MPa)	R^2^
σ_y_	Linear regression	8.26	0.93	17.96	0.84
	Regression trees	16.87	0.71	24.09	0.72
	Ensemble of trees	1.72	0.99	13.79	0.91
	Gaussian process regression	1.65	0.99	25.74	0.67
	Fine Gaussian SVR	8.99	0.86	18.31	0.83
	Artificial neural networks	1.65	0.99	7.14	0.96
	Linear regression	8.65	0.89	16.44	0.55
	Regression trees	10.96	0.84	6.91	0.92
	Ensemble of trees	1.85	0.99	10.09	0.83
σ_u_	Gaussian process regression	1.68	0.99	21.22	0.26
	Fine Gaussian SVR	9.90	0.87	16.28	0.56
	Artificial neural networks	1.68	0.99	5.74	0.95
	Linear regression	1.22	0.90	2.57	0.45
	Regression trees	2.27	0.66	1.23	0.87
	Ensemble of trees	0.23	0.99	1.73	0.75
D_u_	Gaussian process regression	0.30	0.99	3.06	0.23
	Fine Gaussian SVR	1.79	0.79	2.45	0.50
	Artificial neural networks	0.22	0.99	0.93	0.93

**Table 5:** Evaluation metrics of I_T_ machine learning models for both the training and testing sets.

	Training Set		Testing Set	
ML Algorithm	RMSE (%)	R^2^	RMSE (%)	R^2^
Linear regression	7.33	0.84	10.75	−0.04
Regression trees	12.66	0.52	8.38	0.36
Ensemble of trees	1.57	0.99	17.11	−1.64
Gaussian process regression	1.16	0.99	11.49	0.0048
Fine Gaussian SVR	7.99	0.81	11.76	−0.25
Artificial neural networks	1.16	0.99	2.41	0.95

**Table 6:** Evaluation metrics of E_C_ machine learning models for both the training and testing sets.

	Training Set		Testing Set	
ML Algorithm	RMSE (%IACS)	R^2^	RMSE (%IACS)	R^2^
Linear regression	7.33	0.84	10.75	−0.04
Regression trees	12.66	0.52	8.38	0.36
Ensemble of trees	1.57	0.99	17.11	−1.64
Gaussian process regression	1.16	0.99	11.49	0.0048
Fine Gaussian SVR	7.99	0.81	11.76	−0.25
Artificial neural networks	7.99	0.81	2.41	0.97

## Data Availability

All the raw data supporting the conclusion of this paper were provided by the authors.
